# Integrating cancer genomic data into electronic health records

**DOI:** 10.1186/s13073-016-0371-3

**Published:** 2016-10-26

**Authors:** Jeremy L. Warner, Sandeep K. Jain, Mia A. Levy

**Affiliations:** 1 Department of Medicine, Division of Hematology/Oncology, Vanderbilt University, Nashville, TN USA; 2 Department of Biomedical Informatics, Vanderbilt University, Nashville, TN 37232 USA; 3 Vanderbilt-Ingram Cancer Center, Vanderbilt University Medical Center, Nashville, TN 37232 USA; 4 Vanderbilt University School of Medicine, Nashville, TN 37232 USA

## Abstract

The rise of genomically targeted therapies and immunotherapy has revolutionized the practice of oncology in the last 10–15 years. At the same time, new technologies and the electronic health record (EHR) in particular have permeated the oncology clinic. Initially designed as billing and clinical documentation systems, EHR systems have not anticipated the complexity and variety of genomic information that needs to be reviewed, interpreted, and acted upon on a daily basis. Improved integration of cancer genomic data with EHR systems will help guide clinician decision making, support secondary uses, and ultimately improve patient care within oncology clinics. Some of the key factors relating to the challenge of integrating cancer genomic data into EHRs include: the bioinformatics pipelines that translate raw genomic data into meaningful, actionable results; the role of human curation in the interpretation of variant calls; and the need for consistent standards with regard to genomic and clinical data. Several emerging paradigms for integration are discussed in this review, including: non-standardized efforts between individual institutions and genomic testing laboratories; “middleware” products that portray genomic information, albeit outside of the clinical workflow; and application programming interfaces that have the potential to work within clinical workflow. The critical need for clinical-genomic knowledge bases, which can be independent or integrated into the aforementioned solutions, is also discussed.

**Electronic supplementary material:**

The online version of this article (doi:10.1186/s13073-016-0371-3) contains supplementary material, which is available to authorized users.

## Background

The practice of oncology has increased dramatically in complexity since the first chemotherapeutic, nitrogen mustard, was used in 1942. This complexity began with the introduction of combination chemotherapy in the late 1960s and increased significantly with the development of selective “targeted” therapies designed to impair mutated proteins. As treatments have evolved, so too has the understanding of the genetic underpinnings of cancer, which has led to the burgeoning field of cancer genomics [1–4]. However, on a fundamental level, genomics does not alter the paradigm of clinical cancer medicine, of which the cornerstones remain prevention, diagnosis, prognosis, treatment, monitoring, and re-treatment. Rather, genomic data offer the opportunity to refine each of these essential activities of clinical care.

An aspect of cancer care that cannot be overlooked is the importance of proper clinical documentation. The treatment of cancer is a team effort requiring good communication among a diverse team (for example, medical doctors, radiologists, surgeons, pathologists, nurse practitioners, primary care physicians, and others). Electronic health records (EHRs) serve as one vital method through which these team members can coordinate their care. Cancer patient EHRs are complex, due to inherently complicated patient histories, important family histories, detailed social histories, large numbers of testing and imaging results, extensive treatment histories, and cancer genomic information. Unfortunately, there is no current standard for how EHRs should be structured, although they are heavily influenced by the concept of the problem-oriented medical record [5]. Similarly, there is no set method for integrating cancer genomic data into the EHR. For a more thorough review of EHRs and their role in clinical documentation, see the position paper by the Medical Informatics Committee of the American College of Physicians [6].

Here, we first briefly review the general role that genomics plays in each of the fundamental areas of clinical cancer medicine and the current state of cancer genomics through the diverse range of genomic tests that are available today. We then address the current state of integrating cancer genomic data into patient EHRs and review emerging efforts to hone this integration.

## Genomics in clinical cancer medicine

### Prevention

It has been projected that somewhere between 40 % to 50 % of cancers can be prevented if our current research on risk factors is implemented perfectly as public health measures [7]. Some of these methods include proper use of oncogenic virus vaccinations, tobacco exposure control, use of screening guidelines, and elimination of carcinogens from the immediate environment. Clearly, precision medicine—for example, as envisioned by US President Obama’s Precision Medicine Initiative (PMI) [8]—including cancer genomic information, will play a major role in cancer prevention. Mutation profiles may be used more regularly to help stratify patients in need of more rigorous screening protocols [7]. Another interesting area of work involving cancer prevention is the thorough analysis of tumor microenvironments (TMEs). There are now known TME epigenetic regulators and genetic drivers that can be used to elucidate individualized information regarding tumor prevention; there are ongoing efforts to create the Pre-Cancer Genome Atlas (PCGA) to better portray such genomic information for cancer prevention [9]. There is currently no standard documentation of cancer prevention measures within a patient’s EHR. As a “pre-cancer genome atlas” is developed, it will be important to incorporate this information into EHRs to help document individualized preventative measures.

### Diagnosis

The cancer diagnosis is usually straightforward and established on the basis of histology, sometimes with extremely limited material. However, genomics does have a role in certain areas of cancer diagnosis. Sarcomas, which are often de-differentiated, can be subtyped successfully through molecular signatures [10, 11]. Cancer of unknown primary, a wastebasket diagnosis that previously included up to 10 % of metastatic cancer, may become a relic of the past with tissue-of-origin molecular profiling [12]. Molecular analogs, such as BRCA-like ovarian cancer and BCR-ABL1-like acute lymphoblastic leukemia (ALL), have been elucidated through a combination of gene expression and molecular profiling techniques [13, 14]. Histological findings are typically entered into an ancillary laboratory information system as pathology reports. Depending on the center of care, these reports are either scanned into the EHR or entered electronically through an interface. Molecular signatures and molecular profiling reports are provided by the companies that perform them and are usually scanned in as separate reports (PDF format) within EHR systems or kept as hard copies in patients’ files. Few institutions currently have a process by which this information is imported electronically into the EHR system.

### Prognosis

Prognosis depends critically on both cancer biology and host fitness—i.e., performance status and the presence of comorbidity. Obviously, measures of somatic gene aberration can only address the former, whereas clinical judgment remains the determinant of the latter. Staging also remains chiefly anatomic at this time, although biomarkers, which are indirect measures of genetic aberration, have been incorporated into the staging of prostate and testicular cancers with the 7th edition of the American Joint Committee on Cancer (AJCC) Staging Manual [15]; more biomarkers are expected with the 8th edition, to be published in late 2016. Despite this paradigm, some of the earliest correlations between genomic information and phenotype were in the area of prognosis. Long before the genes responsible were characterized, it was recognized that certain karyotypic abnormalities were associated with relatively good or poor prognosis in acute myeloid leukemia (AML) [16–18]. More recently, structural variation and point mutations have been found to have prognostic value, at times independent from any other measurable clinical factor, in most cancers (see for example, [19]). Such information is often entered into a patient’s EHR as a separate report or addendum. If the information is relevant to a patient’s prognosis, as in the case of specific AML subtypes, it is up to the clinician to seek out this information and include it manually in clinical notes.

### Treatment

The recognition that some, if not all, cancers are oncogene-addicted led to the quest for genomically targeted treatments. Many such treatments were discovered before the mechanism for their effectiveness was recognized, such as gefitinib and mutated epidermal growth factor receptor (EGFR) in lung adenocarcinoma [20–22]. The first treatment proactively designed to destroy cells reliant on an aberrant oncogene was imatinib, targeting the fusion protein BCR-ABL [23]. Nearly contemporaneously, imatinib was shown to be an effective KIT inhibitor and to be useful for the treatment of KIT-mutated gastrointestinal stromal tumor, as well [24]. This important recognition that targeted therapies can have multiple modes of “actionability” has led to a complex and promising ecosystem of targeted treatments and the guidance of their selection by molecular profiling panels (see below). Recently, genomics has also begun to identify candidates for immunotherapy, although these approaches do not yet have clinical application [25]. Treatments are entered into patient EHRs by several methods. They may be found as orders that have been placed by the clinician. Prior and current treatments may also be found within narrative clinical notes.

### Monitoring

With few exceptions characterized by durable responses (for example, chronic myelogenous leukemia [CML] treated with imatinib or other tyrosine kinase inhibitors now carries a life expectancy approaching that of age-matched controls [26]), most oncogene-driven cancers recur or progress under the pressure of targeted therapy [27]. For some that can be observed directly for genomic evolution, disease status can be monitored through measurement of the quantity or character of the target protein. For example, lack of response to tyrosine kinase inhibition, as measured by log-reduction in *BCR-ABL1* transcripts, is now a provisional criterion for accelerated-phase CML in the 2016 revision to the World Health Organization classification of myeloid neoplasms and acute leukemia [28, 29]. For the solid malignancies, radiologic monitoring using standard response criteria (for example, Response Evaluation Criteria in Solid Tumors [RECIST]) remains the most common approach [30]. Recently, measurement of the genomes of circulating tumor DNA as a means of monitoring response has gained great interest (see below). Some of these monitoring methods may be entered into a patient’s EHR as laboratory values within a patient’s clinical note. It is also possible that these monitoring methods are reported separately in documents obtained from the testing laboratory.

### Re-treatment

For most cancer types, the evidence base for relapsed and refractory treatment has been both weaker and more diffuse than the evidence base for initial treatment—weaker because there are far fewer published randomized controlled trials, and more diffuse in the sense that many trials in these settings, especially for heavily pretreated patients, allow patients who have received a diversity of prior treatments, making cross-patient comparison more difficult. Some oncogene-addicted cancers will have stereotyped genomic escape mechanisms, leading to progression (for example, ABL kinase domain mutations in CML [31] and gain of EGFR p.T790M mutation in EGFR-mutated lung adenocarcinoma [32]). When such mechanisms are identified, next-generation treatments can be developed, such as ponatinib for CML with ABL p.T315I mutation [33] and osimertinib and rociletinib for non-small-cell lung cancer with EGFR p.T790M mutation [34, 35]. However, these scenarios are likely to be the exception, not the rule. For example, Johnson et al. [36] have shown a diversity of escape mechanisms in vemurafenib-resistant BRAF-mutated melanoma. Increasingly, clinical trial eligibility, such as for the NCI-MATCH trial [37], requires confirmation of the presence or absence of certain mutations. Thus, treatment selection at the time of progression will likely require extensive genomic analysis in most cases.

## Current status of genomic and related information

Integrating genomic information into EHRs may have many interesting results. Understanding these impacts necessitates a brief review of the current and emerging technologies used to represent genomic data clinically. The scope of genomic and related test information that *could* be present in EHRs is large and growing. Most of these data are currently concatenated and duplicated within clinical notes, and are produced by a combination of local and third-party laboratory facilities. Table 1 provides a contemporary list of technologies used in cancer care, which are also summarized briefly here:

**Table 1:** Current status of genomic and related information

Technologies	Applications	Challenges
IHC	Measuring gene overexpression	Expensive
Flow cytometry	Cell surface protein tagging by fluorophores, detects co-expression and loss of expression	Limited spectral frequencies of fluorophores
FISH	Copy number and rearrangement detection	Only works on known targets, cannot detect novel aberrations
Polymerase chain reaction	Confirmatory test and detection of minimal residual disease	May only be scaled to a limited number of variants
Gene expression panels	Production of a single score based on gene expression panel	Commercially available products are based on older datasets
NGS panels	Detection of somatic variants using mostly full-exon sequencing. NGS panels may vary greatly in size (25–500+ genes)	Removing spurious results, identifying VUS, presenting results to clinicians
WES/WGS	Sequencing of coding/all DNA, respectively	High cost, computational complexity, handling VUS, handling incidental findings
Circulating cell-free tumor DNA	Monitoring solid tumor heterogeneity, surveying difficult-to-reach tumors	Not yet widely accepted, no consensus on technical approach, slow turnaround, high cost
Washable IHC	Measuring protein expression with limited tissue sampling	Expensive technique, still experimental
Mass cytometry	Protein tagging by metal ion tags, detects co-expression and loss of expression	Only applicable in cases with known targets, expensive, still experimental
Methylation panels	Determines methylation patterns, which correlate with hypomethylating agent efficacy	Slow adoption of these panels

*FISH* fluorescence in situ hybridization, *IHC* immunohistochemistry, *NGS* next-generation sequencing, *VUS* variants of unknown/uncertain/undetermined significance, *WES* whole-exome sequencing, *WGS* whole-genome sequencing

**Immunohistochemistry**: Includes hundreds of stains available mostly to measure (over)-expression, but some are characteristic of an underlying translocation (for example, *ALK* rearrangement [38]). They are expensive, require dedicated slides, and as such are usually hand-selected by pathologists, such that there are typically 5–10 results per case before tissue is exhausted.

**Flow cytometry**: Measures the expression of cell surface proteins by tagging them with fluorophores. Usually used to characterize hematologic cancers by looking at protein co-expression, as well as loss of expression. Conventional flow is limited by the spectral frequencies of fluorophores such that there are usually four or eight channels. Interpretation involves comparing two-dimensional scatterplots of one channel versus another channel [39].

**Fluorescence in situ hybridization**: Looks for copy number variation and rearrangements. Usually a single test (for example, *ERBB2*/*HER2* amplification testing) or a limited panel of approximately five tests (for example, chronic lymphocytic leukemia panel, myeloma panel). Two related technologies, array comparative genomic hybridization (aCGH) and molecular inversion probe-based (MIP) array, may have more utility in the testing of solid tumors [40, 41].

**Polymerase chain reaction**: Used to confirm certain diseases (for example, CML) and also to detect minimal residual disease to very small scales. Although the turnaround is relatively fast, PCR can only be scaled to “hotspot” testing of 40–50 variants, such as the SNaPshot test [42].

**Gene expression panels**: Used by a limited number of commercial laboratories, such as Genomic Health’s Oncotype DX® platform and Agendia’s MammaPrint® assay. The commercial vendors typically produce a single score from a gene expression panel, and do not make the individual contributing results available external to their laboratory. While these tests can be clinically useful, they are based on older datasets; for example, MammaPrint’s gene expression panel is based on 14-year-old data [43, 44].

**Next**-**generation sequencing panels**: This testing is carried out on tumor tissues and, occasionally, on a comparison of tumor and adjacent normal tissue. Generally, the panels include full exon sequencing and limited intronic sequencing of a panel of genes implicated in the prognosis or treatment prediction of cancers [45]. These range from focused panels of 25–30 genes for a particular cancer subtype to upwards of 500 genes for the largest panels. The three main challenges in next-generation sequencing (NGS) are: 1) removing spurious results, such as those arising from rare germline variants; 2) identifying variants of unknown significance (VUS) and determining their pathogenicity; and 3) presenting results to clinicians. Much of the discussion below will pertain to NGS panels.

There are also several emerging technologies that are likely to be available for clinical care in the next 3–5 years:

**Whole**-**exome sequencing and whole**-**genome sequencing**: Whole-exome sequencing (WES) seeks to characterize the 3 % of coding DNA in a cancer, whereas whole-genome sequencing (WGS) seeks to sequence all DNA [46, 47]. WES and WGS will likely be most useful for determining factors that may indicate response to immunotherapy, such as predicted formation of neoantigens [48]. While these techniques also offer a highly accurate measure of mutational burden, it has recently been shown that NGS panels may suffice for this [49, 50]. Tarczy-Hornoch et al. [51] have surveyed potential methods for properly integrating WES and WGS information within EHRs. Such integration would greatly help with active clinical decision support (CDS).

**Circulating cell**-**free tumor DNA sequencing**: One emerging technology most likely to make inroads into the clinic soon is the analysis of circulating cell-free tumor DNA (ctDNA). Early results have shown that the technology is feasible and reasonably concordant with tissue-based assays [52]. As such, ctDNA can be used as a “liquid biopsy” and help survey complicated cases involving metastatic and difficult-to-reach tumors [53]. Given the ease of specimen collection, this will be used increasingly, especially for solid tumors, as an alternative or replacement for tumor-based genomic testing. Even beyond the ease of specimen collection, ctDNA may play a major role in monitoring solid tumor heterogeneity. NGS of solid tumor samples is limited by tumor sampling bias. The small portion of the tumor biopsied for sequencing likely does not capture the true heterogeneity of the entire solid tumor. Free of this “solid biopsy” sampling bias, ctDNA advances can help to better capture tumor heterogeneity and, therefore, pre-existing or emergent resistance mechanisms [54].

**Washable immunohistochemistry**: New methods are being developed where an immunohistochemistry (IHC) stain can be applied and then removed, followed by another IHC stain on the same slide. This removes the prior limitation of IHC, which is the availability of stainable material (for example, a cell block made from pleural fluid sampling can support the creation of only five to six unstained slides; similar limitations exist for fine-needle aspirations) [55]. It remains to be seen whether costs will support high-dimensional IHC testing.

**Mass cytometry**: Mass cytometry is a variation of flow cytometry in which antibodies are labeled with heavy metal ion tags, rather than fluorochromes, and has the potential to replace conventional flow cytometry [56]. Readout is by time-of-flight mass spectrometry. This technology can measure tens or hundreds of parameters and is being actively evaluated for subtyping AML and other leukemias [57].

**Methylation panels**: There are only two commercially available hypomethylating agents, decitabine and azacytidine. Their exact mechanism of efficacy is unknown but is under active investigation. It appears that alterations in methylation patterns in noncoding DNA are likely responsible for the observed efficacy, and presumably, there will be tests developed to predict for the efficacy of these and other antineoplastic agents, although progress in this field has been disappointingly slow [58].

## Integration of genomic data into the EHR: Current status

Contemporary to the rise of genomics in most aspects of clinical cancer care, EHRs have become ubiquitous, through a combination of “meaningful use” regulations and the expected diffusion of innovations [59–61]. With the wealth of genomic data now available to inform various aspects of cancer care, the casual observer will be surprised to discover that only a small minority of this information is incorporated into the EHR in a format amenable to electronic search, CDS, or secondary use, despite some hopeful predictions made a decade ago [62–64]. Instead, many genomic tests, especially multiplex panels, are reported in PDF format and are either physically mailed or faxed to the ordering provider. This practice stems from several factors, including: 1) bioinformatics pipelines intervening between raw data and clinical reporting of variants, 2) the role of human curation in the interpretation of variant calls, and 3) lack of consistent standards for the transfer of genomic laboratory results. Each of these factors is discussed below, and the reader is also referred to the experience of the Electronic Medical Records and Genomics Network (eMERGE), summarized by Kho et al. [65].

### Bioinformatics pipelines

No matter the technology to measure somatic gene variation, an extensive processing pipeline is required to transform raw data into meaningful information; this differentiates genomic testing from the majority of routine clinical laboratory testing. While a comprehensive review of such pipelines is beyond the scope of this article and is provided elsewhere [66, 67], two points are worth noting, regarding the interrelated concepts of 1) variant calling and 2) coverage.

The first point is the challenge of variant calling—that is, distinguishing detected variants from normal germline variation. This challenge pervades the pipeline process, from sequence alignment to single-nucleotide variant (SNV) calls, and is a concern for all areas of genetic testing, not just cancer-specific testing, as illustrated by a recent case study by Manrai et al. [68]. Although some laboratories undertake tumor-normal testing to identify and remove germline variants [69], this technique roughly doubles the cost of the test, and insurers have balked at covering the increased cost [70]. Given the presence of somatic genomic variants in “normal” tissue (for example, benign acquired melanocytic nevi are enriched for *BRAF* mutations [71]), this approach also runs the risk of type II errors (that is, false negatives). Current practices for tumor-only testing dictate the use of a reference database, such as 1000 Genomes [72] or ExaC [73], usually augmented by a reference laboratory’s locally hosted proprietary knowledge. The concept of a normal reference human genome is undergoing evolution and will likely be replaced by the concept of genome graphs, which does away with the idea of a single reference genome and replaces it with a diversity of genomes based on graph theory [74, 75].

The second challenge is in regards to coverage—that is, the need to obtain a statistically reliable signal. Most NGS reads will not be identical, because the starting and ending base pairs are not the same. The majority of reads will have a single error, but with multiple reads, it is likely that many will be identical to at least one other read [45]. For a given sequence, the number of times that a sequence is read is referred to as the read depth; across all sequences for a given test, summary statistics, such as the average (mean) depth of coverage, are critical quality assurance data for laboratories and are sometimes reported. Read depths are not uniform across the genome and may not even be parametric; as a result, statistics, such as the mean, do not appropriately capture the reliability of the test. Due in part to the fact that this information is often kept private by laboratories, in July 2016, the US Food and Drug Administration (FDA) proposed draft guidance, entitled “Use of Standards in FDA Regulatory Oversight of Next-Generation Sequencing (NGS)-Based In Vitro Diagnostics (IVDs) Used for Diagnosing Germline Diseases” [76]. As the title indicates, this draft guidance is aimed towards germline testing, not somatic variant testing. However, the suggestions are still informative. With regard to coverage, the FDA proposes the following: “For detecting germline heterozygous variants using a targeted panel, set a threshold of 20× or greater for minimum coverage depth and 300× for average coverage depth at 100 % of the bases for targeted panels and at least 97 % of the bases for WES.”

To address these challenges, guidelines have been issued by the US Centers for Disease Prevention and Control (CDC) [77], the New York State Department of Health (updated in 2016) [78], and the American College of Medical Genetics and Genomics (ACMG) [79], but none of them is likely to have the impact that the FDA guidance will have on the regulation of NGS bioinformatics pipelines.

### Interpretation of results

Transforming raw genomic *data* into somatic variant call *information* is the first step that is necessary for clinical interpretation, but is not in and of itself sufficient. In order to act on this information, it must be transformed into meaningful clinical *knowledge*. It has become readily apparent that the majority of cancers have thousands, if not hundreds of thousands, of discrete mutations, most of which are nonfunctional and related to background mutations, genomic instability, or defects in the neoplastic DNA repair machinery [80–82]. Because of this, the concept of “clinical actionability” has gained currency, and *ad hoc* definitions of this phrase have emerged over time (see Table 2). The main challenge for incorporating “clinical actionability” into the EHR is two-fold: 1) explaining actionability, especially when multiple variants are detected, usually requires lengthy prose with multiple literature references and 2) actionability is subject to change as new information becomes known. The importance of consistent interpretation of variant results is illustrated by an ongoing lawsuit (*Williams v Quest/Athena*), in which it has been alleged by the plaintiff that an *SCN1A* variant was reported as a VUS but was later determined to be pathogenic [83], and the results of the Prospective Registry of MultiPlex Testing (PROMPT) study, which has demonstrated a large incidence of discordance across genetic testing laboratories [84].

**Table 2:** An example of an actionability hierarchy for identified genomic variants

Hierarchical level^a^	Example scenario^b^
1. Variant *known* to confer sensitivity to an FDA-approved agent for the cancer subtype	1. BRAF p.V600E mutation 2. Vemurafenib 3. Melanoma
2. Variant *predicted* to confer sensitivity to an FDA-approved agent for the cancer subtype	1. BRAF p.V600K mutation 2. Vemurafenib 3. Melanoma
3. Variant *known* to confer sensitivity to an FDA-approved agent for *another* cancer subtype	1. BRAF p.V600E mutation 2. Vemurafenib 3. Hairy cell leukemia
4. Variant *predicted* to confer sensitivity to an FDA-approved agent for *another* cancer subtype	1. BRAF p.V600K mutation 2. Vemurafenib 3. Lung adenocarcinoma
5. Variant *known* to confer sensitivity to an experimental agent for the cancer subtype	1. BRAF p.V600E mutation 2. Binimetinib 3. Melanoma
6. Variant *known* to confer sensitivity to an experimental agent for *another* cancer subtype	1. BRAF p.V600E mutation 2. Binimetinib 3. Hairy cell leukemia
7. Variant *predicted* to confer sensitivity to an experimental agent for the cancer subtype	1. BRAF p.V600K mutation 2. Binimetinib 3. Melanoma
8. Variant with *known* prognostic significance for the cancer subtype	1. KMT2A rearrangement t(4;11)(q21;q23) as sole abnormality 2. B-cell ALL 3. Poor prognosis in adults
9. Variant with *predicted* prognostic significance for the cancer subtype	1. ABL1 p.M244V mutation 2. CML 3. Likely poor prognosis, faster progression to accelerated or blast phase
10. VUS	1. BRCA1 p.S645Y mutation 2. Triple-negative breast cancer 3. No known sensitivity or prognostic significance

*ALL* acute lymphoblastic leukemia, *CML* chronic myeloid leukemia, *FDA* Food and Drug Administration, *VUS* variant of unknown significance

^a^Hierarchy of actionability of identified genomic variants, ranging from the situation with the strongest evidence base relating cause and effect (for example, treatment of the given condition with a given drug will result in an expected response) (1) to the weakest (10). For each hierarchical level, an example is provided that meets three criteria: 1) genomic variant, 2) pharmacologic agent, and 3) disease context. For simplicity, we do not further delineate disease context by status (for example, untreated, relapsed/refractory), although pharmaceutical agents are increasingly FDA-approved only for a given disease context *and* status

^b^The examples use predicted sensitivity but predicted resistance has the equivalent hierarchy

### Lack of consistent standards

Perhaps the greatest challenge to the integration of genomic laboratory results into EHRs has been the lack of consistent standards for the unambiguous transfer of such information [85, 86]. While there are well-established nomenclatures for the representation of genetic variation, such as HUGO Gene Nomenclature Committee (HGNC) for gene names [87], Human Genome Variation Society (HGVS) for SNVs and indels [88], and International System for Human Cytogenetic Nomenclature (ISCN) for structural variation [89], applying these nomenclatures with vigor has not yet occurred in the clinical domain. As a simple example, consider the FDA label for the BRAF inhibitor vemurafenib: “for the treatment of patients with unresectable or metastatic melanoma with BRAFV600E mutation as detected by an FDA-approved test.” The character string “BRAFV600E” is neither HGNC- nor HGVS-compliant; yet, this type of result is often seen in the PDF reports issued by molecular laboratories (personal communication, Mollie Ullman-Cullere, Better Outcomes). Instead, “BRAF c.1799 T > A (p.Val600Glu)” or simply “BRAF p.V600E” would be compliant. Although the distinction may seem minor, the downstream implications for integrated CDS, interoperability, and secondary data use are significant [90]. Beyond the use of appropriate nomenclature, standard representation of unambiguous facts, such as “sensitivity to vemurafenib,” is problematic. The issue is not a paucity of standards but, rather, too many to choose from (see Table 3 and Additional file 1: Tables S1 and S2). A decision to bind to a non-widely accepted or insufficiently granular terminology can have major downstream effects and hamper interoperability, especially when clear translations between terminologies are not readily available. In an attempt to counter this outcome, the Health Level Seven International (HL7®) Clinical Genomics work group developed a fully Logical Observation Identifiers Names Codes (LOINC)-qualified genetic variation implementation guide for HL7 Version 2 (V2) messaging, updated in 2013 [91]. However, this approach has not been widely adopted, in part because V2 does not excel in capturing the richness of a prose document, such as that required for the interpretation of the results. A more contemporary effort, called “V2-lite,” is currently underway at HL7, and the Fast Healthcare Interoperability Resources (FHIR®) approach is increasingly promising (see below).

**Table 3:** Terminology systems that uniquely identify the genomically targeted antineoplastic drug vemurafenib^a^

Terminology short name	Terminology long name (if applicable)	Definition	Unique code	Website
ATC	Anatomical Therapeutic Chemical classification system	vemurafenib	L01XE15	http://www.whocc.no/atc_ddd_index/
CAS Registry Number	Chemical Abstracts Service Registry Number	vemurafenib	918504-65-1	http://ww.cas.org/content/chemical-substances
ChEBI	Chemical Entities of Biological Interest	vemurafenib	CHEBI:63637	https://www.ebi.ac.uk/chebi/
ChEMBL		vemurafenib	CHEMBL1229517	https://www.ebi.ac.uk/chembl/
ChemSpider		vemurafenib	24747352	http://www.chemspider.com
DrugBank		vemurafenib	DB08881	http://www.drugbank.ca
eMolecules		vemurafenib	32176418	https://www.emolecules.com
FDA UNII Code	Food and Drug Administration Unique Ingredient Identifier	vemurafenib	207SMY3FQT	https://fdasis.nlm.nih.gov/srs/srs.jsp
Guide to Pharmacology	IUPHAR/BPS Guide to Pharmacology	vemurafenib	5893	http://www.guidetopharmacology.org
InChI	IUPAC International Chemical Identifier	vemurafenib	GPXBXXGIAQBQNI-UHFFFAOYSA-N	https://iupac.org/who-we-are/divisions/division-details/inchi/
KEGG DRUG	Kyoto Encyclopedia of Genes and Genomes	vemurafenib	D09996	http://www.genome.jp/kegg/drug/
MeSH	Medical Subject Headings	PLX4032	C551177	https://www.ncbi.nlm.nih.gov/mesh
NCI Thesaurus	National Cancer Institute Thesaurus	vemurafenib	C64768	https://ncit.nci.nih.gov/ncitbrowser/
NCI-GLOSS	NCI Dictionary of Cancer Terms	PLX4032	CDR0000670004	https://www.cancer.gov/publications/dictionaries/cancer-terms
PDBe	Protein Data Bank in Europe	PLX4032	32	http://www.ebi.ac.uk/pdbe/
PDQ	Physician Data Query	vemurafenib	CDR0000528954	https://www.cancer.gov/publications/pdq
PubChem		vemurafenib	CID:42611257	https://pubchem.ncbi.nlm.nih.gov
RxNorm		vemurafenib	RxCUI:1147220	https://www.nlm.nih.gov/research/umls/rxnorm/
SNOMED-CT_US	Systematized Nomenclature of Medicine - Clinical Terms, US Realm	Vemurafenib (product)	SCTID:703656005	https://www.nlm.nih.gov/healthit/snomedct/us_edition.html
UMLS	Unified Medical Language System	vemurafenib	C1832009	https://www.nlm.nih.gov/research/umls/
ZINC		vemurafenib	ZINC52509366	http://zinc15.docking.org

^a^While these 21 distinct terminologies may not be exhaustive, they do illustrate the challenge of using terminology bindings in standards. Similar complexity is observed in terminologies for diseases, genes, proteins, and pathways (see Additional file 1)

## Integration of genomic data into the EHR: Emerging solutions

While the current status quo of faxed reports scanned into EHRs is mostly acceptable for individual patient care, it does not allow for CDS or secondary use of data. Several emerging paradigms illustrate how genomic data may be more thoroughly integrated into EHRs and clinical workflows in the near future. We will review non-standardized integration approaches, “middleware,” application programming interfaces (APIs), efforts to create standardized EHR applications, and emerging knowledge bases. Non-standardized integration will allow integration of genomic information into EHRs with limited to no interoperability between institutions. “Middleware” creates a platform that is not fully integrated with an institution’s EHR system; however, it has been shown to be a useful modality for conveying up-to-date genomic information to clinicians. APIs have the potential of being fully integrated within a clinician’s workflow; however, standardization of genomic concepts is a necessary first step towards this reality.

**Non**-**standardized integration into EHRs**: One solution is to create a custom interface between a third-party genomics laboratory and a freestanding EHR installation. The advantage of this approach is that it can be implemented relatively quickly. The disadvantage is that it is not readily extendable to other laboratories or EHRs. In 2014, Vanderbilt University Medical Center developed such an interface with Foundation Medicine Inc., and we at that center can now receive electronic results of the FoundationOne test on a real-time basis. In addition to preservation of fidelity in the transmission, this interface automatically matches results to patients and notifies providers when the test results are ready through their existing notification workflow [92]. We did find errors occurring, mostly attributable to mismatches in free-text fields, such as medical record number and patient name; these errors were mostly correctable through provider education. In addition to incorporation into the clinical EHR, the results are shared with a research and operations database, which has enabled secondary use for clinical trial feasibility and cohort identification needs [93]. Non-standardized integration therefore allows genomic information to be viewed within a clinician’s existing workflow while simultaneously facilitating research endeavors.

### Middleware

Recognizing that the user’s needs were not being met, several products have emerged that can be loosely termed “middleware,” comprising standalone web portals or platforms for displaying patient cancer genomic data. The most common of these products are web portals provided by third-party laboratories. Examples of such products would be Foundation Medicine’s ICE portal [94] and Caris Life Science’s MI Portal [95]. These products have two main disadvantages: 1) they are not within clinician workflow and typically require a separate login, and 2) they have limited ability to merge clinical data with genomic data, without further data entry on the part of the ordering clinician. On the other hand, they will contain high-fidelity results, which can be updated as new knowledge accumulates, and often contain links to primary literature and clinical trials resources. Another class of middleware products is exemplified by Syapse Inc., which produces a “platform” that serves several needs related to cancer genomics: workflow management, including ordering and receiving results; integration of clinical and molecular data; CDS; and support for activities, such as molecular tumor boards. Several leading organizations, such as UCSF and Intermountain Healthcare, are currently implementing the Syapse platform [96]. However, this solution is likely to be unaffordable to community oncologists, as it requires expensive manual integration, and it is limited by the degree to which clinical information can be shared by the host EHR. While “middleware” does not handle clinical information very well, it fills the much needed gap of portraying patient genomic information, albeit outside of the clinician’s workflow.

**Application programming interfaces**: The power of using a standardized encoded representation of genomic data becomes most evident when taking advantage of the emerging complementary ecosystem of APIs, applications (apps), and third-party knowledge bases [97]. As a simple example, the Physician Data Query (PDQ) identification code for vemurafenib (CDR0000528954, from Table 3) can be entered directly into the URL of the NCI Drug Dictionary [98] so as to return the appropriate page describing the drug, with links out to active clinical trials. A more complicated example of this representational state transfer (RESTful) approach to web services is the OpenFDA API [99], which will take a variety of coded representations and return a list of reported adverse events to a given pharmacologic agent, in XML structure. This structure can then be transformed for user presentation through any of a variety of apps. Importantly, OpenFDA and similar APIs that expose non-patient health information data usually require a thin layer of security, in the form of uniquely identifiable API keys.

### APIs in the medical domain

**Fig. 1:**
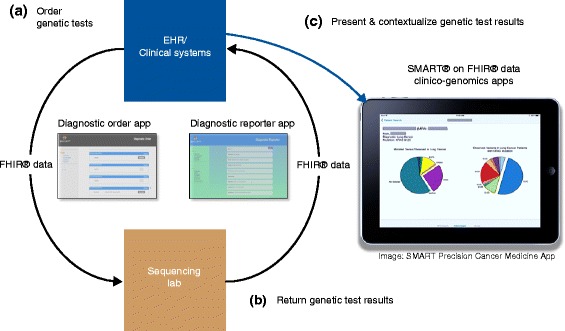
FHIR Genomics can be used to enable multiple steps in the genomic testing and interpretation process. The figure shows a hypothetical workflow that a clinician would carry out. **a** First, any of a number of genetics tests are ordered electronically, and the details are transmitted to an internal or third-party lab, for example a sequencing lab. This step can be accomplished using an app such as the Diagnostic Order App or through native electronic health record (EHR) capabilities. **b** Second, the lab generates structured test results which are returned to the clinician within their workflow. This step can be accomplished using an app such as the Diagnostic Reporter App or through direct interfaces. **c** Third, results can be presented and contextualized for the clinician at the point of care through apps that can integrate clinical and genomic data, such as SMART Precision Cancer Medicine. Figure courtesy of David Kreda

Apps that operate within the clinical domain require stricter authorization and security procedures. The Substitutable Medical Apps, Reusable Technologies (SMART®) platform was developed to enable the existence of such apps, which can, importantly, be launched from within or external to an EHR [97, 100]. SMART applications therefore have the potential to be used within the clinical workflow, including the ability to work on tablet devices and to support single sign-on authority. Towards the end of the initial SMART grant, the HL7 FHIR standard began to gain momentum. As a result, the SMART platform was modified to take advantage of FHIR, and the result was SMART on FHIR [101]. FHIR operates on the concept of a group of core “resources” meant to capture the bulk (around 80 %) of information present in current EHRs and to provide ready means to extend the standard to capture the other 20 % (including genomic data). We demonstrated that such an extension was possible for genomic data in the SMART on FHIR environment [102] and subsequently developed a prototype app that could display population-level genomic data in the context of an individual patient, SMART Precision Cancer Medicine (PCM, Fig. 1) [103]. More recently, the concept of a sequence (for example, DNA, protein) has been brought into the core FHIR resources [104]. As FHIR captures an increasing number of concepts from EHR systems, the possibility of ubiquitous SMART applications increases, allowing patients to take such applications from institution to institution.

The PMI, which aims to collect biospecimens and EHR data from at least 1 million participants [8], has further galvanized the development of the SMART on FHIR effort, specifically through a related initiative, called “Sync 4 Science” [105]. This initiative, which is intended to establish an ongoing feed between an EHR and the PMI Cohort Program database, involves the placement of an app with revocable long-term authorization within a patient portal and is actively undergoing implementation by seven large EHR vendors [106]. Encouragingly, a recent survey demonstrated a broad willingness to share data and samples for the PMI Cohort Program, and this consumer engagement is likely to push the integration of EHR and genomic data even more rapidly [107].

## Utilizing genomic data in the EHR: The need for knowledge bases

In parallel with the evolution of apps, free and commercial knowledge bases have begun to emerge to capture the complexity of the marriage of genomic and clinical data. One of the earliest publicly available knowledge bases, MyCancerGenome [108], was started in 2011 and now has information on 22 cancer types and 823 cancer genes [109, 110]. More recently, the Jackson Laboratory has released a semi-automated/manually curated database of disease, variant, drug, and clinical trial relationships for 82 genes (as of October 2016): the JAX-Clinical Knowledgebase [111, 112]. Another database with similar application is the OncoKB database, developed and maintained by Memorial Sloan Kettering in partnership with Quest Diagnostics [113]. This knowledge base contains information about the treatment implications of specific cancer gene variants and goes on to classify treatment information based on a Levels of Evidence system. Clinical Interpretation of Variants in Cancer (CIViC) is yet another knowledge database that captures variant-level cancer genomic information [114]. The mission of the open-source, open-access CIViC knowledge base is primarily education and dissemination of information that has been curated by community users and domain experts.

Clarivate Analytics (formerly the Intellectual Property and Science business of Thomson Reuters) has released a subscription-only product, Precision Medicine Intelligence, that is manually curated, with information on 8514 genes and 89,631 genetic variants (including intergenic SNPs and structural variants), as they relate to drug sensitivity, clinical trials, and prognosis (personal communication, Melinda Baker, Clarivate Analytics). This product also employs a 12-point evidence scoring algorithm, which assesses the clinical applicability of a variant association through a combination of effect sizes, strength of correlations, reproducibility, and the statistical rigor used in the source publication(s). More general than cancer, the ClinGen genomic knowledge base [115] is intended to be an “authoritative central resource that defines the clinical relevance of genes and variants for use in precision medicine and research” [116, 117]. The live portal was recently opened, and there is an active EHR working group, whose task is to “ensure that the ClinGen resource is designed to be accessible to providers and patients through electronic health record and related systems.”

With this proliferation of knowledge bases, it can be difficult to choose among them. Recognizing this swiftly changing ecosystem, the FDA issued a partner draft guidance document to the one referred to previously, entitled “Use of Public Human Genetic Variant Databases to Support Clinical Validity for Next-Generation Sequencing (NGS)-Based In Vitro Diagnostics” [76]. The goal of the eventual guidance is to provide oversight for publicly accessible databases that are providing aggregation, curation, and/or interpretation services.

**Fig. 2:**
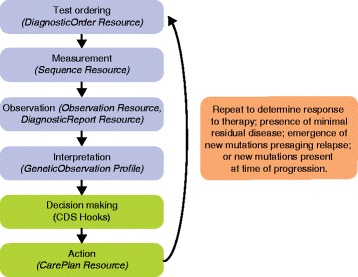
Genomic information in the flow of cancer care. This simplified flow diagram illustrates the process of information gathering and decision making that characterizes the standard model of interventional oncology care. In particular, this model is applicable to the treatment, monitoring, and re-treatment phases of oncology care. In blue are primarily the information gathering steps, and in green are the active decision making and intervention steps. This process is inherently iterative, usually on a pre-planned schedule such as assessment of treatment response after 8 weeks of therapy, or surveillance monitoring on a quarterly basis. Each step of this process can be captured by one or more FHIR Resources/Profiles, which are shown in italics in parentheses. CDS Hooks is a special implementation of FHIR for clinical decision support purposes (see text for details)

One can easily see the scenario where an app, either within or external to an EHR, can “hook” into an external knowledge base to provide information at the point of care. The pilot implementation of PCM demonstrated seamless linkages to three knowledge bases: Gene Wiki, MyCancerGenome, and HemOnc.org [104]. Eventually, guidance in the form of genomic CDS could also be offered through apps. Such “CDS hooks” would be synchronous to the clinical workflow and would only launch when needed and are under active development [118]. The eMERGE and Implementing GeNomics In Practice (IGNITE) consortia have also produced a knowledge base of genomic medicine CDS artifacts [119]. An example of a genomically informed clinical workflow is shown in Fig. 2.

## Conclusions

The avenues for obtaining cancer genomic information have increased rapidly and will continue to do so as the costs of genomic testing go down and insurance reimbursement becomes more routine. From NGS to methylation panels, we have vast amounts of information characterizing myriad cancer types and their sensitivities to treatment. While the oncologic data grow in both size and sophistication, the basics of patient care remain largely unchanged. Today’s major challenge is to make the complicated cancer genomic data compatible with our more traditional clinician-patient interactions. A useful first step in addressing this challenge is to solve the problem of cancer genomic data integration with EHRs.

By having cancer genomic information available in EHRs, providers and patients both stand to benefit, especially with the movement to more openly shared EHRs [120]. Perhaps patients would be better informed as to why they are receiving certain more expensive targeted antineoplastic medications as opposed to cheaper nonselective alternatives. Perhaps patients could better understand why their prognosis has changed after receiving a particular genomic test. In other words, cancer genomic information integration into EHRs could help promote the benefits of patient-centered care.

Beyond upholding clinician-to-patient interactions, integrating cancer genomic information with EHRs could be a major driver of scientific discovery. Substantial amounts of useful clinical data are available in the long oncologic narratives within EHRs. Having that information side by side with genomic cancer information could help unveil correlations and patterns that were previously obscure.

An interesting area of development that will undoubtedly harness cancer genomic-EHR integration will be machine learning algorithms and CDS software. Machine learning algorithms will be better able to identify patterns in patients’ genomic and clinical data, enlightening clinicians on information and associations that may have been overlooked. CDS mechanisms will one day be able to augment the ability of doctors to shape treatment courses. It is important to note that the development and maintenance of CDS are not free and may exacerbate disparities, if the appropriate ethical frameworks are not considered in advance. We anticipate that this important discussion, including whether the benefits justify the costs, will need to take place sooner rather than later.

Having cancer genomic information integrated into EHRs will undoubtedly help clinicians take better care of patients. With proper integration, patients and their cancer genomic information should be able to travel more seamlessly between care centers; we have previously shown that such interoperability is possible [121]. Other projects, such as the National Academy of Medicine’s DIGITizE [122], are also working on integrating genetic information into the EHR. Furthermore, clinicians may be more inclined to let the genomic information in their patients’ EHRs better guide the decisions they make if it is well integrated. For example, well-integrated cancer genomic information within an EHR could inform doctors of other patients with similar variants and their course of therapy. On the other hand, such integration of genomic information in EHRs could help clinicians realize why their patient is unique from the populations described to date in clinical trials and case studies. In essence, proper integration would help take the practice of medicine towards the future of personalized and precision medicine.

## Additional file

Additional file 1: Table S1. Nomenclatures of relevance to genomic medicine. **Table S2.** Terminology systems that uniquely identify genes, proteins, or pathways. (DOCX 32 kb)

## Abbreviations

AML: acute myeloid leukemia
API: application programming interface
CDS: clinical decision support
CML: chronic myeloid leukemia
ctDNA: circulating cell-free tumor DNA
EHR: electronic health record
FDA: Food and Drug Administration
FHIR: Fast Healthcare Interoperability Resources
HL7: Health Level Seven International
IHC: immunohistochemistry
NGS: next-generation sequencing
PGCA: Pre-Cancer Genome Atlas
PMI: Precision Medicine Initiative
SMART: Substitutable Medical Apps, Reusable Technologies
V2: HL7 Version 2 messaging
VUS: variants of unknown/uncertain/undetermined significance
WES: whole-exome sequencing
WGS: whole-genome sequencing
