# Supplementary material for: Integrating cancer genomic data into electronic health records
Source: Genome Med. 2016 Oct 26;8:113. doi: 10.1186/s13073-016-0371-3 (PMC5081968; doi:10.1186/s13073-016-0371-3)
Supplement: Additional file 1: Table S1. — Nomenclatures of relevance to genomic medicine. Table S2. Terminology systems that uniquely identify genes, proteins, or pathways. (DOCX 32 kb) [file 13073_2016_371_MOESM1_ESM.docx]

**Additional file 1**

| **Table S1 Nomenclatures of relevance to genomic medicine** | | | |
| --- | --- | --- | --- |
| **Nomenclature short name** | **Nomenclature long name** | **Example** | **Website or reference** |
| HGVS | Human Genome Variation Society | BRAF p.V600E | http://www.hgvs.org/mutnomen/ |
| HLA | Nomenclature for Factors of the HLA System | HLA-A*02:101:01:02N | http://hla.alleles.org/nomenclature/index.html |
| HLA GL String | HLA Genotype List String | A*02:69+A*23:30\|A*02:302+A*23:26/A*23:39 | Milius et al., 2013 [1] |
| ISCN | International System for Human Cytogenetics Nomenclature | t(11;17) | McGowan-Jordan et al., 2016 [2] |
| Star allele | The Human Cytochrome P450 (CYP) Allele Nomenclature | CYP3A5*3K | http://www.cypalleles.ki.se/ |

| **Table S2 Terminology systems that uniquely identify genes, proteins, or pathways. This list is not intended to be exhaustive and primarily includes databases that are freely accessible** | | | | |
| --- | --- | --- | --- | --- |
| **Terminology short name** | **Terminology long name (if applicable)** | **Example** | **Unique code** | **Website or reference** |
| Gene terminologies | | | | |
| CBO | Clinical Bioinformatics Ontology | (gDNA).*BRAF* | CLINBIO!ASnztQEMgk5hj4AXCr0EUA | Hoffman et al., 2005 [3] |
| CCDS | Consensus Coding Sequence Project | *BRAF* | CCDS5863.1 | <https://www.ncbi.nlm.nih.gov/CCDS/CcdsBrowse.cgi> |
| CGAP | Cancer Genome Anatomy Project | Hs. *BRAF*, V-raf murine sarcoma viral oncogene homolog B1 | CID:550061 | [http://cgap.nci.nih.gov](http://cgap.nci.nih.gov/) |
| COSMIC | Catalogue Of Somatic Mutations In Cancer | *BRAF* | COSG2 | <http://cancer.sanger.ac.uk/cosmic> |
| Ensembl |  | *BRAF* | ENSG00000157764.10 | <http://www.ensembl.org/index.html> |
| GenBank |  | Homo sapiens v-raf murine sarcoma viral oncogene homolog B1 (*BRAF*) gene, complete cds | EU600171.1 | <https://www.ncbi.nlm.nih.gov/genbank/> |
| Gene | Entrez Gene | Human *BRAF* | 673 | <https://www.ncbi.nlm.nih.gov/gene/> |
| GeneCards |  | V-Raf Murine Sarcoma Viral Oncogene Homolog B | GC07M140424 | [http://www.genecards.org](http://www.genecards.org/) |
| H-InvDB | H-Invitational Database | Serine/threonine-protein kinase B-raf | HIX0007148 | [http://www.h-invitational.jp](http://www.h-invitational.jp/) |
| HGNC | Human Gene Nomenclature Committee | *BRAF* | HGNC:1097 | [http://genenames.org](http://genenames.org/) |
| KEGG GENES | Kyoto Encyclopedia of Genes and Genomes | Human *BRAF* | hsa:673 | <http://www.genome.jp/kegg/genes.html> |
| LRG | Locus-Reference-Genomic | *BRAF* | LRG_299 | [http://www.lrg-sequence.org](http://www.lrg-sequence.org/) |
| NCI Thesaurus | National Cancer Institute Thesaurus | *BRAF* Gene | C18363 | <https://ncit.nci.nih.gov/ncitbrowser/> |
| NCI-GLOSS | NCI Dictionary of Cancer Terms | *BRAF* gene | CDR0000561325 | <https://www.cancer.gov/publications/dictionaries/cancer-terms> |
| OMIM | Online Mendelian Inheritance in Man | V-RAF MURINE SARCOMA VIRAL ONCOGENE HOMOLOG B1; *BRAF* | *164757 | <https://www.ncbi.nlm.nih.gov/omim> |
| PharmGKB | Pharmacogenomics Knowledgebase | *BRAF* | PA25408 | [https://www.pharmgkb.org](https://www.pharmgkb.org/) |
| RefSeqGene |  | Homo sapiens B-Raf proto-oncogene, serine/threonine kinase (*BRAF*), mRNA | NM_004333.4 | <https://www.ncbi.nlm.nih.gov/refseq/rsg/> |
| RefSeqNucleotide |  | Homo sapiens v-raf murine sarcoma viral oncogene homolog B (*BRAF*) | NG_007873.3 | <https://www.ncbi.nlm.nih.gov/nuccore/> |
| UCSC Genome Browser |  | Human Gene *BRAF* | uc003vwc.4 | <https://genome.ucsc.edu/> |
| UMLS | Unified Medical Language System | *BRAF* Gene | C0812241 | <https://www.nlm.nih.gov/research/umls/> |
| UniGene |  | V-raf murine sarcoma viral oncogene homolog B1 (*BRAF*) | Hs.550061 | <https://www.ncbi.nlm.nih.gov/unigene> |
| Vega |  | *BRAF* | OTTHUMG00000157457 | <http://vega.sanger.ac.uk/index.html> |
| Gene alteration terminologies | | | | |
| DbSNP | Single-Nucleotide Polymorphism Database | rs17822931 | rs17822931 | <https://www.ncbi.nlm.nih.gov/projects/SNP/> |
| DbVar | Database of genomic structural variation | copy number variation involving *BRAF* | esv3615243 | <https://www.ncbi.nlm.nih.gov/dbvar> |
| LSDB | Locus-specific Database | *Multiple, depending on the individual LSDB* | *Depends on the individual LSDB* | <http://grenada.lumc.nl/LSDB_list/lsdbs> |
| DbSNP | Single-Nucleotide Polymorphism Database | rs17822931 | rs17822931 | <https://www.ncbi.nlm.nih.gov/projects/SNP/> |
| ClinVar |  | NM_004333.4(*BRAF*):c.1799T>A (p.Val600Glu) | 13961 | <https://www.ncbi.nlm.nih.gov/clinvar/> |
| COSMIC | Catalogue Of Somatic Mutations In Cancer | BRAF p.V600E / c.1799T>A | COSM476 | <http://cancer.sanger.ac.uk/cosmic> |
| Gene linked to another concept terminologies | | | | |
| KEGG ORTHOLOGY | Kyoto Encyclopedia of Genes and Genomes | *BRAF* | K04365 | <http://www.genome.jp/kegg/ko.html> |
| OMIM | Online Mendelian Inheritance in Man | LEOPARD SYNDROME 3; LPRD3 | #613707 | <https://www.ncbi.nlm.nih.gov/omim> |
| Orphanet |  | *BRAF* - B-Raf proto-oncogene, serine/threonine kinase | ORPHA119066 | <http://www.orpha.net/> |
| Protein terminologies | | | | |
| ChEMBL |  | Serine/threonine-protein kinase B-raf | CHEMBL5145 | <https://www.ebi.ac.uk/chembl/> |
| Guide to Pharmacology | IUPHAR/BPS Guide to Pharmacology | B-Raf proto-oncogene, serine/threonine kinase | 1943 | [http://www.guidetopharmacology.org](http://www.guidetopharmacology.org/) |
| HPRD | Human Protein Reference Database | B-Raf | HPRD:01264 | [http://www.hprd.org](http://www.hprd.org/) |
| IntAct | An open source molecular interaction database | braf_human | EBI-365980 | <http://www.ebi.ac.uk/intact/> |
| InterPro |  | Serine/threonine-protein kinase B-raf | P15056 | <https://www.ebi.ac.uk/interpro/> |
| LOVD 3.0 | Leiden Open Variation Database | v-raf murine sarcoma viral oncogene homolog B1 | 03475 | <http://www.lovd.nl/> |
| PDB | RCSB Protein Data Bank | Serine/threonine-protein kinase B-raf - P15056 (BRAF_HUMAN) | P15056 | <http://www.rcsb.org/pdb/home/home.do> |
| PhosphoSitePlus |  | BRAF (human) | P15056 | <http://www.phosphosite.org/> |
| RefSeq Protein |  | serine/threonine-protein kinase B-raf [Homo sapiens] | NP_004324.2 | <https://www.ncbi.nlm.nih.gov/refseq/> |
| UniProt |  | Serine/threonine-protein kinase B-raf | P15056 | [http://www.uniprot.org](http://www.uniprot.org/) |
| Protein alteration terminologies | | | | |
| ExPASy | Expert Protein Analysis System | UniProtKB/Swiss-Prot P15056: Variant p.Val600Glu | VAR_018629 | <https://www.expasy.org/> |
| NCI Thesaurus | National Cancer Institute Thesaurus | BRAF V600E Mutation Present | C80275 | <https://ncit.nci.nih.gov/ncitbrowser/> |
| OMIM | Online Mendelian Inheritance in Man | MELANOMA, MALIGNANT, SOMATIC BRAF, VAL600GLU | *164757.0001 | <https://www.ncbi.nlm.nih.gov/omim> |
| UMLS | Unified Medical Language System | BRAF V600E Mutation Present | C2698302 | <https://www.nlm.nih.gov/research/umls/> |
| Protein pathway terminologies | | | | |
| Pfam |  | Protein tyrosine kinase | PF07714 | [http://pfam.xfam.org](http://pfam.xfam.org/) |
| BioSystems | NCBI BioSystems | MAPK cascade | BSID: 921026 | <https://www.ncbi.nlm.nih.gov/Structure/biosystems/docs/biosystems_about.html> |
| Gene Ontology |  | MAPK cascade | GO:0000165 | [http://geneontology.org](http://geneontology.org/) |
| KEGG PATHWAY | Kyoto Encyclopedia of Genes and Genomes | MAPK signaling pathway | hsa04010 | <http://www.genome.jp/kegg/pathway.html> |
| Reactome |  | Signaling by FGFR | REACT_9470.8 | [http://www.reactome.org](http://www.reactome.org/) |
| WikiPathways |  | MAPK cascade | WP1343 | http://wikipathways.org/index.php/WikiPathways |
| KEGG MODULE | Kyoto Encyclopedia of Genes and Genomes | MAPK (ERK1/2) signaling | hsa_M00687 | <http://www.genome.jp/kegg/module.html> |

**References**

1. Milius RP, Mack SJ, Hollenbach JA, Pollack J, Heuer ML, Gragert L et al (2013) Genotype List String: a grammar for describing HLA and KIR genotyping results in a text string. Tissue Antigens. 82:106–112.

2. McGowan-Jordan J, Simons A, Schmid M. An International System for Human Cytogenomic Nomenclature. Basel: Karger AG; 2016.

3. Hoffman M, Arnoldi C, Chuang I (2005) The clinical bioinformatics ontology: a curated semantic network utilizing RefSeq information. Pac Symp Biocomput. 139–150.
